# Prevalence of Nontuberculous Mycobacteria in Cystic Fibrosis Clinics, United Kingdom, 2009

**DOI:** 10.3201/eid1907.120615

**Published:** 2013-07

**Authors:** Paul Seddon, Katy Fidler, Sundhya Raman, Hilary Wyatt, Gary Ruiz, Caroline Elston, Felicity Perrin, Khin Gyi, Diana Bilton, Francis Drobniewski, Melanie Newport

**Affiliations:** Royal Alexandra Children’s Hospital, Brighton, UK (P. Seddon, K. Fidler);; Brighton and Sussex Medical School, Brighton (K. Fidler, S. Raman, M. Newport);; Kings College Hospital, London, UK (H. Wyatt, G. Ruiz, C. Elston, F. Perrin);; Royal Brompton Hospital, London (K. Gyi, D. Bilton);; Barts and the London School of Medicine and Dentistry, Queen Mary, University of London, London (F. Drobniewski)

**Keywords:** nontuberculous mycobacteria, cystic fibrosis, *Mycobacterium avium* complex, *Mycobacterium absessus* complex, bacteria, United Kingdom, tuberculosis and other mycobacteria

## Abstract

Incidence of pulmonary infection with nontuberculous mycobacteria (NTM) is increasing among persons with cystic fibrosis (CF). We assessed prevalence and management in CF centers in the United Kingdom and found 5.0% of 3,805 adults and 3.3% of 3,317 children had recently been diagnosed with NTM. Of those, 44% of adults and 47% of children received treatment.

Nontuberculous mycobacteria (NTM) are ubiquitous environmental organisms (*1*), broadly classifiable into “slow” and “rapid” growers. Many species, especially the slow-growing *Mycobacterium avium* complex (MAC), are known to cause disseminated disease in immunodeficient persons (*2*,*3*). Rapid growers include the phylogenetically similar *M. abscessus* and *M. chelonae*, referred to as *M. abscessus* complex (MABSC). Such organisms have emerged as pathogens in immunocompetent adults, for example, after traumatic limb injuries sustained during the 2004 tsunami in the Indian Ocean (*4*). NTM have been identified as pulmonary pathogens in immunocompetent middle-aged women with nodular bronchiectasis (*5*) and older men who smoke with upper lobe cavitation (*6*). Since the 1990s, NTM have been increasingly isolated from the sputum of patients with cystic fibrosis (CF) (*7*,*8*).

CF is the most frequent lethal genetic disorder of White persons, affecting >8,000 persons in the United Kingdom and 30,000 in the United States. Early death is mainly from chronic lung disease caused by persistent lower airway infection and inflammation. Important airway pathogens include *Staphylococcus aureus* and *Pseudomonas aeruginosa*, but others, such as NTM, are playing an increasingly recognized role.

A multicenter prospective study of CF patients in the United States (*9*) found the prevalence of NTM in sputum to be 13%; MAC was the most common species (72%), and *M. abscessus* was the next most common (16%). Older age was the most significant predictor for a positive sputum culture. A multicenter CF study in France reported a prevalence of 6.6%, showing MABSC to be the most common; MAC was the next most prevalent (*10*). Single-center studies in Europe have found variable NTM prevalences, from 13.3% in a center in Germany (*7*) to 3.8% in a center in the United Kingdom (*8*).

It is often difficult to determine whether isolation of NTM represents colonization or disease that requires treatment. Current American Thoracic Society guidelines (*1*) are helpful, but application lacks uniformity, and management is complicated by inducible resistance to antibiotics (*11*).

To determine the optimal management of NTM in CF, we first need to define the extent of the problem and characterize current practice. The aim of this study was to establish the prevalence of NTM infection in the UK CF community, how it is screened for, and how it is managed.

## The Study

A single-page questionnaire (Technical Appendix) was sent to the lead CF physician in all UK pediatric and adult CF centers identified by the principal UK CF charity, the Cystic Fibrosis Trust, in 2009. Nonresponders were followed up with by email or telephone. The local research ethics committee deemed the study did not require ethical approval.

Results were tabulated and basic statistical analysis performed by using Microsoft Excel 2007 (Microsoft, Redmond, WA, USA). Because *M. chelonae and M. abscessus* have common phenotypic characteristics and not all samples were fully sequenced by a reference laboratory, we combined these for reporting as MABSC.

Twenty-three adult and 29 pediatric UK CF centers, with 8,513 CF patients, were sent questionnaires. Responses were received from 19 (83%) and 24 (83%) of these centers respectively, comprising 7,122 patients (3,805 adults, 3,317 children), or 84% of the UK CF population. As expected, the prevalence of any NTM isolate was higher in the adult (5.0%) than in the pediatric (3.3%) population (Table). Fifteen adult and 17 pediatric centers cultured samples for NTM yearly; most remaining centers tested for NTM only if there was a specific clinical indication. The NTM pathogens most frequently cultured were the rapidly growing MABSC (62% of adults, 68% of children who were NTM-positive), followed by MAC (28% of adults, 27% of children). Other species (*M. gordonae, kansasii, xenopi, fortuitum, simiae, malmoense, mucogenicum, perigrinum*) together made up 8% of the total. Wide geographic variation was noted, increasing from northwest to southeast, manifesting lowest prevalence in Northern Ireland (1.9%) and highest in South East England (7.5%) (Figure).

**Table,:** NTM isolation rate and screening frequency among adult and pediatric cystic fibrosis clinics, United Kingdom, 2009

CF clinic type	No. clinics receiving/no. responding to survey (%)	Total no. patients	No. (%) clinics		Frequency of requesting NTM culture
			With any NTM isolates in previous 2 y	With >2 NTM isolates in previous 2 y	
Adult	19/23 (83)	3,805	190 (5.0)	135 (3.6)	Yearly: 15
					Every 6 months: 1
					Every 3 months 1
					When clinically indicated: 2
Pediatric	24/29 (83)	3,317	110 (3.3)	74 (2.2)	Yearly: 17
					Every 6 months: 0
					Every 3 months 0
					When clinically indicated: 7

*NTM, nontuberculous mycobacteria.

**Figure:**
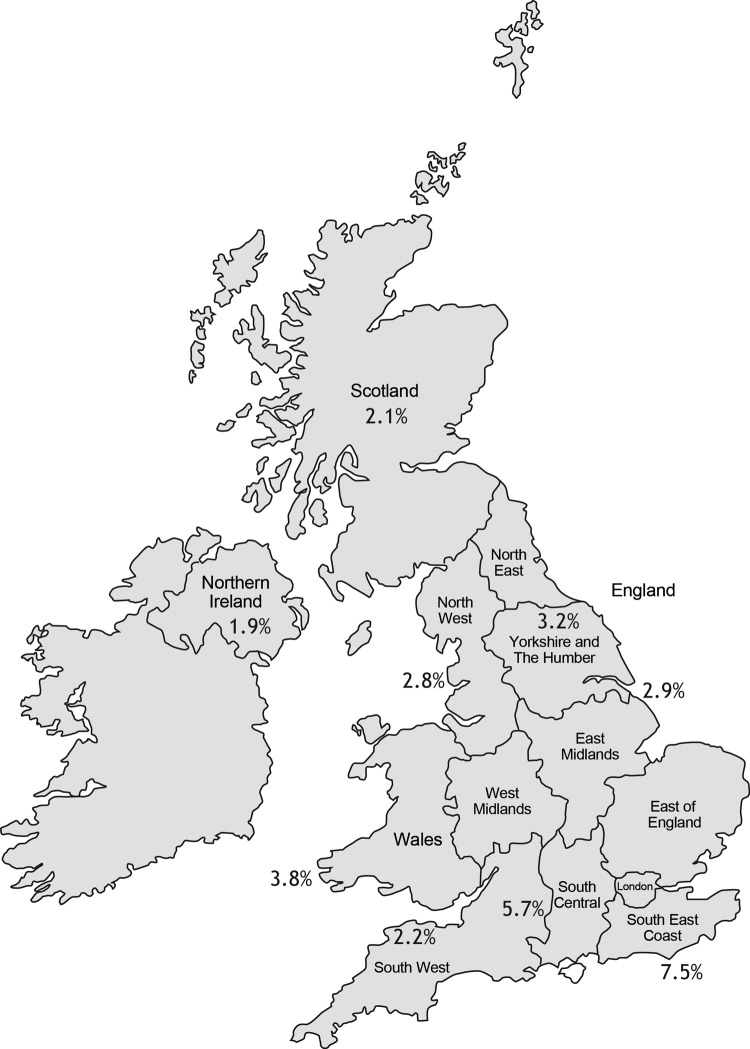
Regional variation in percentage of cystic fibrosis patients in whom nontuberculous mycobacteria were isolated, United Kingdom, 2009. Percentages represent combined clinics within participating regions. Nineteen of 23 adult and 24 of 29 pediatric CF centers, accounting for 7,122 of 8,513 (84%) CF patients, participated in the survey.

Criteria for starting treatment varied widely between centers. Most centers required clear clinical or radiologic deterioration in addition to positive cultures, but a significantly higher proportion of adult centers (42%) than pediatric centers (21%) were prepared to commence treatment on the basis of repeated culture or smear positivity alone. Despite this, similar proportions of NTM-positive adults and children had received specific treatment for NTM in the 2 years preceding the survey: 44% of adults and 47% of children with any NTM isolate were treated, and 62% of adults and 70% of children in whom >2 isolates were found were treated. Six adults and 2 children who had CF had been refused a lung transplant on the basis of persistent NTM infection.

Our results differ from a 2003 multicenter US survey (*9*) in 2 crucial ways. First, the overall prevalence of NTM of 4.2% during the study period in the United Kingdom was much lower than the 13% reported for the study done in the United States. This could be caused in part by lower sampling frequency, occurring annually in most UK clinics, compared with 3 times annually as reported in the US study. The proportion of patients having >2 positive cultures was similar: 3.9% for the US study and 3.0% for our study. Furthermore, the denominator used for prevalence estimation may have been overestimated in this study: it may have included some patients missed for annual culture; and particularly for pediatric clinics, the denominator would include non–sputum producers (cough swabs are normally not cultured for NTM). The questionnaire we used did not specify which source documents respondents used; therefore, recall bias may be an issue. However, all UK centers extract and report NTM data to a national database annually.

The second striking difference between this study and the US study was that rapid-growing mycobacteria (especially MABSC) predominated in this survey while MAC predominated in the US study. Our results are more in keeping with subsequent European and Middle East prevalence studies (*7*,*10*,*12*). Rapid-growing mycobacteria such as MABSC are now recognized to be of greater clinical importance in CF than MAC (*1*), and a recent US single-center survey (*13*) has shown *M. abscessus* to be the current dominant NTM and to be associated with evidence of clinical decline.

As in previous studies, we found marked geographic variation in prevalence: Olivier et al. found higher rates in coastal centers in the United States (*9*), and Roux et al. found the highest prevalence in west and southwest France (*10*). The higher prevalence in the south of the United Kingdom could be caused by different sampling rates, but we found no systematic difference in screening policy between different UK regions. Climatic differences are relatively small across a small temperate island such as the United Kingdom. Geology could be another factor; NTM acquisition has been linked with water from aquifers (*12*,*14*) which are present mainly in the younger rocks in southeastern United Kingdom.

The varied threshold for instigating NTM treatment is perhaps not surprising in view of the lack of evidence to guide this. Although there is no clear evidence to justify treatment on positive cultures alone, some centers may have lowered their threshold in the hope that (by analogy with *Pseudomonas aeruginosa*) aggressive early treatment might lead to eradication. Refusal of lung transplantation is clearly an issue and appears to affect the threshold for treatment, though the rationale for refusal by transplant centers remains controversial (*15*).

## Conclusions

This survey highlights the growing importance of NTM as a CF pathogen, the importance of routine, standardized surveillance, and the need to find out how best to manage NTM in CF. This will require further research into environmental, microbial, and host factors influencing acquisition and disease progression of NTM in the CF population.

Technical Appendix Questionnaire: a survey of nontuberculous mycobacteria in patients with cystic fibrosis.
