# Supplementary material for: Prevalence of Nontuberculous Mycobacteria in Cystic Fibrosis Clinics, United Kingdom, 2009
Source: Emerg Infect Dis. 2013 Jul;19(7):1128–30. doi: 10.3201/eid1907.120615 (PMC3713964; doi:10.3201/eid1907.120615)
Supplement: Technical Appendix — Questionnaire: a survey of nontuberculous mycobacteria in patients with cystic fibrosis. [file 12-0615-Techapp-s1.pdf]

Article DOI: http://dx.doi.org/10.3201/eid1907.120615

Prevalence and Management of Nontuberculous Mycobacteria in Cystic Fibrosis Clinics, United Kingdom

Technical Appendix

A Survey of Non-Tuberculous Mycobacteria (NTM) in Patients with Cystic Fibrosis (CF)

Letter accompanying survey:

We are a group of clinicians, microbiologists and immunologists carrying out a survey on the prevalence and clinical importance of NTM infections in the CF population in the UK. This is the first part of a stream of research looking at NTM infections in these patients. Many CF units have been increasingly concerned about NTM over the last few years, but there is little consensus on when and how to treat. We would like to enlist your help in shedding some light on this difficult area, by providing some basic information on your patients. We would be very grateful if you could spend a few minutes completing this brief questionnaire, and return it to us either by email or in the enclosed reply-paid envelope (we have sent it out simultaneously by both routes). Alternatively our researcher could contact you to arrange a convenient time to obtain your responses via telephone – if you would prefer this option please respond as such via email.

Many thanks in anticipation for your help.

The collaborative NTM in CF Study Group

- **Royal Alexandra Children's Hospital**
- **Brighton & Sussex Medical School**
- **Kings College Hospital**
- **Royal Brompton Hospital**
- **Clinical Immunology, Addenbrookes Hospital**
- **Mycobacterial Reference Unit, London**

A Survey of Non-Tuberculous Mycobacteria (NTM) in patients with Cystic Fibrosis (CF)

Name of lead consultant	
Name of CF centre	
Centre type (circle)	Paediatric / adult
Number of CF patients	
How frequently are your patients specifically cultured for NTM? (circle)	Yearly / 6-monthly / 3-monthly / Other (specify)
Number of patients who have cultured NTM in the past 2 years	
Number of patients who have cultured NTM on at least 2 separate occasions (each at least 2 weeks apart)	
Number of patients who have cultured:<br>*Mycobacterium avium* complex	
M. Chelonae / M. abscessus	
Other (please specify)	
What would be your criteria for starting treatment for NTM?	2 or more isolates of NTM?<br>2 or more isolates + smear positive?<br>2 or more isolates + smear positive + clinical deterioration ?<br>Other (please specify)
Number of patients on antibiotic treatment for NTM at any stage over the past 2 years	
Number of patients who have been refused a transplant, primarily on the basis of persistent NTM infection, over the past 5 years	
If you have any comments or suggestions you would like to make please add these below	
